# From forced migration to forced arrival: the campization of refugee accommodation in European cities

**DOI:** 10.1186/s40878-017-0069-8

**Published:** 2018-03-28

**Authors:** René Kreichauf

**Affiliations:** 1 Department of Geography, Faculty of Sciences, Vrije Universiteit Brussel, Building F – Room 4.74, Pleinlaan 2, BE-1050 Brussels, Belgium; 2 Graduate School of North American Studies (GSNAS), John-F.-Kennedy Institute for North American Studies, Freie Universität Berlin, Lansstraße 5-9, 14195 Berlin, Germany

**Keywords:** Campization, Refugee camps, Asylum, Urban studies, Arrival infrastructures, Socio-spatial exclusion, Forced migration

## Abstract

In the aftermath of large refugee arrivals in 2015, EU regulations and national asylum laws were tightened, especially those regarding reception and accommodation. The current contribution introduces the concept of “campization” to explain the impact of law and policy changes on the socio-spatial configuration and functions of refugee accommodation in European capital regions. Based on qualitative research concerning case studies for Athens, Berlin, and Copenhagen, I argue that refugee accommodation has increasingly been transformed into large, camp-like structures with lowered living standards and a closed character. This is shown by the structural, functional, and socio-spatial characteristics of the accommodation in the three case studies, as well as the political and administrative objectives that determine the campization of accommodation. The contribution lastly highlights changing notions and forms of containment, exclusion, and temporality as part of campization, and links this process to current trends in asylum and urban development.

## Introduction

In EU member states, large-scale accommodation has become the dominant response to forced migration and the arrival of refugees^1^ (European Migration Network, 2014). Since the 1980s, several states have introduced this into their legal frameworks as the obligatory form of housing migrants with uncertain residence status for the first months–and sometimes years–after their arrival (AIDA, 2016). The concentration of large numbers of refugees in one place is a result of the increasing attempts of the EU and nation states to regulate and reduce the number of refugees arriving (Kreichauf, 2016). In the aftermath of Europe’s refugee crises in 2015, EU regulations and national asylum laws, especially those concerning reception and accommodation, have been tightened.

Here, I argue that changes in legislation have stimulated a transformation of European refugee accommodation in city regions toward large, camp-like structures with lowered housing standards. First, I illustrate that the conceptualization of forced migrants’ accommodation has a global north (“asylum center”) and a global south (“refugee camp”) geography based on literature investigating accommodation practices for refugees in Europe and the genesis of refugee camps in Africa and the Middle East. The current contribution intervenes and brings the camp, theoretically established in literature, to the north. I reveal that concepts and theories of refugee camps, such as demarcation, containment, (legal) exceptionality, temporality, and also agency building, are helpful in understanding the development of camp-like accommodation in Europe.

Second, I introduce the concept of “campization” in order to explain the underlying processes that generate the socio-spatial arrangements of accommodation infrastructures. I describe campization as a process in which the recent tightening of asylum laws and reception regulations have resulted in the emergence and deepening of camp-like characteristics of refugee accommodation in European city regions.

Third, I identify and elucidate these processes and structures through an in-depth comparison of three case studies: The capital regions of Athens, Berlin, and Copenhagen. Based on qualitative research, I make sense of the changes to accommodation structures, asylum laws, and reception regulations, as well as the notions and objectives of exclusion and exception regarding campization. I present the structural, functional, and socio-spatial characteristics that determine campization, and lastly, I link these concepts to current trends in asylum and urban development.

## Theorizing the transformation of refugee accommodation in European cities

The terminology applied to define current mass accommodation for refugees in Europe reveals some conceptual slippage. After the Second World War, this accommodation in Europe was commonly termed *refugee camps*. Even with the introduction of accommodation into the legal asylum frameworks of some European states in the 1980s, it was initially referred to as camps. With the institutionalization of accommodation as part of asylum laws, legal and administrative terms such as (reception and accommodation) *centers*, (asylum) *shelters*, and *homes* have emerged. By signing up to the 1951 Refugee Convention and the European Convention on Human Rights–as well as due to EU directives–EU member states have been obliged to introduce regulations and provisions governing standards of living into their legal frameworks. In studies on institutionalized collective accommodation in and around European cities, researchers thus often refer to “European Accommodation Centres for Asylum Seekers” (Szczepanikova, 2013), “camp-like collective accommodations” (Pieper, 2008), and “asylum centres” (Morville & Erlandsson, 2013). The term “refugee camp” is mostly used to refer to first, camps in the global south, second, discourses on informal encampments, makeshift camps, and tent cities for transiting refugees (such as Calais in France and Idomeni in Greece), and third, hotspots, and detention and transit centers in border areas. This distinction is made to highlight differences in socio-spatial structures, living conditions, the accommodation’s nature, and questions of institutional responsibilities, governing, and labeling spaces and people. In these contexts, camps are referenced as large spaces with an insecure, temporary, or exceptional legal status (for example regarding construction laws), which accommodate masses of people, have low standards of living, and consist of tents, containers, and/or improvised shelters.

Defining refugee accommodation is a struggle of interpretational sovereignty. Witteborn (2011, p. 1149) illustrates that “the practice of naming asylum heterotopias constructed the forced migrant *as* a discursive location.” Pieper (2008, p. 528) explains that labeling these spaces is a result of the political will of those who govern them and of their institutional character. Terms such as asylum center would downplay the objectives of these places and their living conditions. Refugee organizations and refugees themselves predominantly apply the term “refugee camp.” This is because, as Migreurop (2005) and the Alliance against Camps (2014) highlight in an interview, this term not only refers to closed centers with walls, barbed wire, and surveillance devices, but generally to premises that exclude refugees. “Open” sites such as accommodation centers may appear to be designed to provide assistance and shelter, but they have been set up to contain refugees without providing an option other than remaining there. I use the official terms such as reception, emergency, accommodation *center*, etc., when I refer to a specific case. I apply *camp* or the more broad term *accommodation*, when I generally refer to these sites to underline their camp-like characteristics.

In recent decades, much empirical research has been conducted on the exclusion of refugees in European cities through restrictive policies, as well as placement and housing regulations (see e.g., Aumüller, Biesenkamp, & Daphi, 2015; Breckner, 2014; Darling, 2009; Dwyer & Brown, 2008; Hirschler, 2013; Pieper, 2008; Szczepanikova, 2013; Witteborn, 2011). Applying Bourdieu (1991) or Lefebvre (1991), the refugee camp is often analyzed as a space to explain its structures, its socio-spatial nature, and representations of existing power structures (Pieper, 2008). Marx (1990), Harell-Bond (1999), Inhetveen (2014), and Witteborn (2011) apply Auge’s (2008) theory of “non-places,” Goffman’s (1961) total institutions, and/or Foucault’s (1997) heterotopias and biopolitics to conceptualize mass accommodation. In general, researchers studying European accommodation often refer to theories of space as well as to Agamben’s (1998) political philosophy of sovereignty, bare life, and the state of exception.

Many theoretical concepts of the *refugee camp* as a socio-spatial entity were developed based on camps in African countries and the Middle East, most prominently by Agier (2014), Ramadan (2013), Sanyal (2014), and Malkki (1995). In this literature, the refugee camp is usually positioned between “formality and informality, mobility and immobility, permanence and impermanence” (Grbac, 2013, p. 3) as well as exception and norm (Malkki, 2002). Scholars conceptualize these extremes by applying two major theoretical angles. First, camp studies apply an Agambenian and a Foucauldian view to study the camp as a space of exception, biopolitics, and its means of discipline and security. Diken and Laustsen (2005) and Edkins (2000), for example, see camps as spaces that are put into place to control and contain people who “disturb the national order of things” (Turner 2016, p. 139). Hyndman (2000) and Pasquetti (2015) study camps as spaces of discipline, control, order, and governmentality. Second, there are scholars such as Ramadan (2013), Salih (2017), and Redclift (2013), who go beyond the notion of the camp as a representation of a state of exception and exclusion. They follow the approach of studying camps (also) as sites in which new identities, acts of agency, political life, and resistance are formed and practiced. Other works focus on various forms of violence and exploitation (Ferris, 2007; Loescher & Milner, 2004) or conceptualize camps as, or in contrast to, cities (Agier, Nice, & Wacquant, 2002; Grbac, 2013).

Despite several attempts to explain the refugee camp and its functions, there is no coherent definition; the terminological differentiation between European refugee accommodation and non-European camps complicates conceptualizations of camp(−like) housing. The camp itself constitutes a space of contradictions and paradoxes in several respects (Grbac, 2013; McConnachie, 2016). The different approaches not only emerge because of varying disciplinary angles, geographical lenses, epistemological or methodological approaches, or specific research objectives and interests, but also reflect in the end the empirical complexity and structures of the camp as well as different experiences of those “inside.”

Nevertheless, relevant literature provides some common denominators and dimensions, which are applied here to highlight the camp-like features of refugee accommodation in Europe. First, the nature of a camp is to separate populations and to create a distinction between those inside (immigrants as camp residents) and those outside (the local population). Its objective is to contain a specific category of the population. The extent of this containment varies, but segregation is a result of it. Second, camps are demarcated and have boundaries; there is a clear spatial distinction between the space inside and the space outside, physical barriers and other material and social forms of containment (Agier, 2014; McConnachie, 2016). Third, camps are exceptional in legal terms, since they are usually governed by different legal instruments and frameworks than those in the surrounding areas and that apply to citizens of a state (Agamben, 1998). Turner (2016, p. 141) argues that, “they are legally under the jurisdiction of the host society but also exempted from it” due to regulations and laws of asylum and alien acts. Lastly, a refugee camp is a space of permanent temporality. On the one hand, it is not meant to remain; it is not intended to be a durable solution, but is applied as a device to temporarily react to forced migration. On the other hand, the length of the stay in camps and the existence of camps is unknown; they “exist between the temporary and the permanent” (Hailey, 2009, p. 4).

For the study of encampment in European cities, this means that the refugee accommodation must be analyzed within or as part of geographic, political, and social contexts and the transformative nature of these contexts and the space of accommodation itself. A camp is not a static place. It is a socio-spatial process where involved actors, producers, and users, constantly reproduce and transform socio-spatial configurations. To explain the changing patterns of European accommodation and emerging camp features, I apply the concept of campization.

Campization is a process that illustrates two tendencies of accommodating refugees in the context of increasing numbers arriving in EU member states and the tightening of laws on asylum, and explicitly on reception: First, the legal stabilization of permanent, enlarged, remotely located, and spatially isolated camps with lowered living standards, increased capacities, and a closed character; and second, the changing notions and forms of containment, exclusion, and temporality of these infrastructures. These tendencies are reflected architectonically, functionally, and socio-spatially. I present these characteristics of the process of campization later on.

The development of camp-like infrastructures of arrival may also be described as *Forced Infrastructures of Arrival*. State and non-state actor constellations introduced them to territorialize the arrival of refugees in extraterritorial spaces. They have developed into a fundamental instrument and socio-spatial structure in governing the reception of newcomers that facilitates the concentration of refugees in allocated places within an allocated time. They are also increasingly places where asylum laws and policies force refugees to stay for a legally prescribed period, sometimes up to several years.

## Research approach

In what follows here, I present the empirical findings of three cases: Athens Capital Region (Attica), the State of Berlin, and Copenhagen Capital Region (Hovestaden). The research was conducted between May 2013 and July 2016.

Each of the three case studies is the capital and the most diverse region of the country concerned. Accordingly, they are the center for refugee organizations and arriving refugees. However, each plays a different role in the European migration regime, often connected to their location in the North, Middle, and South of Europe. Athens has changed from a city of transition to a city of destination, due to the closing of the Balkan route and the EU-Turkey Statement. Berlin has attracted immigrants and refugees in recent decades. It received around 55,000 refugees in 2015, making it one of the biggest recipients of refugees at the city level in the EU. Hovestaden has a long tradition as a destination for refugees. Denmark was one of the first nations to ratify the 1951 convention, but is known today for its harsh treatment of refugees, its camp policies, and poor living conditions. The access to these cities and their communities is aggravated because of dispersal policies in Denmark and Germany, as well as the remote locations of the accommodation and physically exclusive structures in some cases.^2^

The research first comprises multilevel analyses of major law and policy changes as well as of the development of political and societal discourses on (refugee) immigration in Greece, Germany, and Denmark. The analysis of policies includes research into laws, alien acts, directives, and regulations on three levels: The legislative framework of the EU, national legislations and politics, and local practices and regulations.

Second, the research design includes socio-spatial analyses of eight asylum centers in the regions of Athens, Berlin, and Copenhagen: Elliniko I-III, Eleonas, and Schisto (Athens); Refugium Motardstraße, Refugium Rhinestraße, Emergency Shelter Am Kaiserdamm, and Centre Klingsorstraße (Berlin); Centre Sandholm and Centre Kongelunden (Copenhagen Capital Region).

Lastly, I conducted 34 open and guideline-based interviews concerning the accommodation, living conditions, and the impact of law changes between the following levels: i) decision makers, administrative bodies, and center operators (9 interviews), ii) civil society actors, local refugee organizations, and initiatives (16 interviews), and iii) asylum seekers and refugees (9 interviews). The aim of the three-level division was to develop a broad context of findings concerning the perceptions of social structures, power relations, and effects in and of center housing. The interviews were evaluated using Mayring’s (2011) method of content analyses. Informal talks with the group of refugees were also carried out. They were analyzed as observations as part of the “Go along” method (Kusenbach, 2003).

## Accommodation practices in Athens, Berlin, and Copenhagen

Despite the ongoing implementation of EU regulations into national laws, protection and housing standards differ across member states. The Common European Asylum System (CEAS) provides references to different forms of reception conditions for refugees, including standards, responsibilities, and the management of housing (Council Directive 2003/9/EC). However, they are implemented in different ways in the EU. Some countries have highly developed legal frameworks, integration, housing, and care schemes. There is a clear institutional distinction between first-reception facilities for accommodating new arrivals (first accommodation, registration, and the start of the asylum procedure) and second-line reception for people who have already entered the asylum process (accommodation centers) (AIDA, 2016; European Migration Network, 2014). In other member states, asylum systems and institutional frameworks are still in the process of development. State structures (federal vs. centralized states), the location and role of a member state in Europe (country of transition vs. arrival, Mediterranean states vs. countries in North Europe) and the role of a nation state in EU policymaking processes (initiators vs. receivers, CEAS opt-out states) impact different administrative responsibilities and structures.

In Denmark and Greece, policies concerning housing are made centrally at the nation state level. Denmark is not a part of CEAS and has not fully implemented council directives on the reception, qualification, and asylum procedure. The Immigration Service (a sub-institution of the Ministry of Justice) is responsible for the asylum procedure and housing. It contracts the Danish Red Cross or communes to manage refugee accommodation. In Greece, administrative responsibilities appear to be complicated. Particularly since 2013, it has implemented EU laws creating a stronger legal framework concerning reception and care. The Ministries of Citizen Protection, Migration Policy, and Labour, together with the Greek navy and military are responsible for the management of housing. Often one of these runs the camps directly, or contracts are given to NGOs, which are financed through the European Refugee Fund (ERF). UNHCR and the EU have also initiated an accommodation project, in support of the Greek authorities’ efforts to expand reception capacity since 2014.

In Germany, the Law on Asylum Procedure and the Asylum Seeker’s Benefits Act determine the national organization of housing. The detailed configuration and implementation of housing is the responsibility of the federal states (Länder). Germany introduced an allocation system including quotas for the reception of asylum seekers at the level of its states, the *Königssteiner Schlüssel*. This means that an asylum seeker who arrives in a German state is initially accommodated in a state-run reception center until the distribution scheme allocates her or him to a center in the counties and municipalities of a state that is in charge of the application. As a city state, Berlin’s Senate for Health and Social Affairs and its State Office for Refugee Affairs (LAF) shape Berlin’s housing policy. Consequently, housing patterns in Berlin are shaped by state and local politics, whereas in Copenhagen and Athens they are determined at the national level.

### Athens: From a city of transition to a city of forced destination in large camps

The closing of the Balkan route after the summer of 2015 and the EU-Turkey Statement have changed Athens’ role in managing arrivals. As the capital and largest city of Greece and because of its harbor, Athens attracts refugees stranded in Greece. This new role as a city of forced destination and the shift from state actors’ and civil society’s approaches of short-term assistance in transit to long-term responsibilities has challenged state and city officials to provide broader based support and accommodation (Papagianakis, 2016). Articles 12 and 13 of the Presidential Decree 220/2007 (Proedriko Diatagma [Presidential Decree] 220/2007), guarantee reception, housing, and maintenance in “adequate” accommodation in Greece. Athens’ city government has introduced, for the first time in its history, a department for migration and refugee affairs, an integration policy, and an Immigrant Integration Council, which coordinates the municipality’s actions. However, in practice, resources have not been sufficient for the reception of around 17,000 refugees in Attica.

**Map 1:**
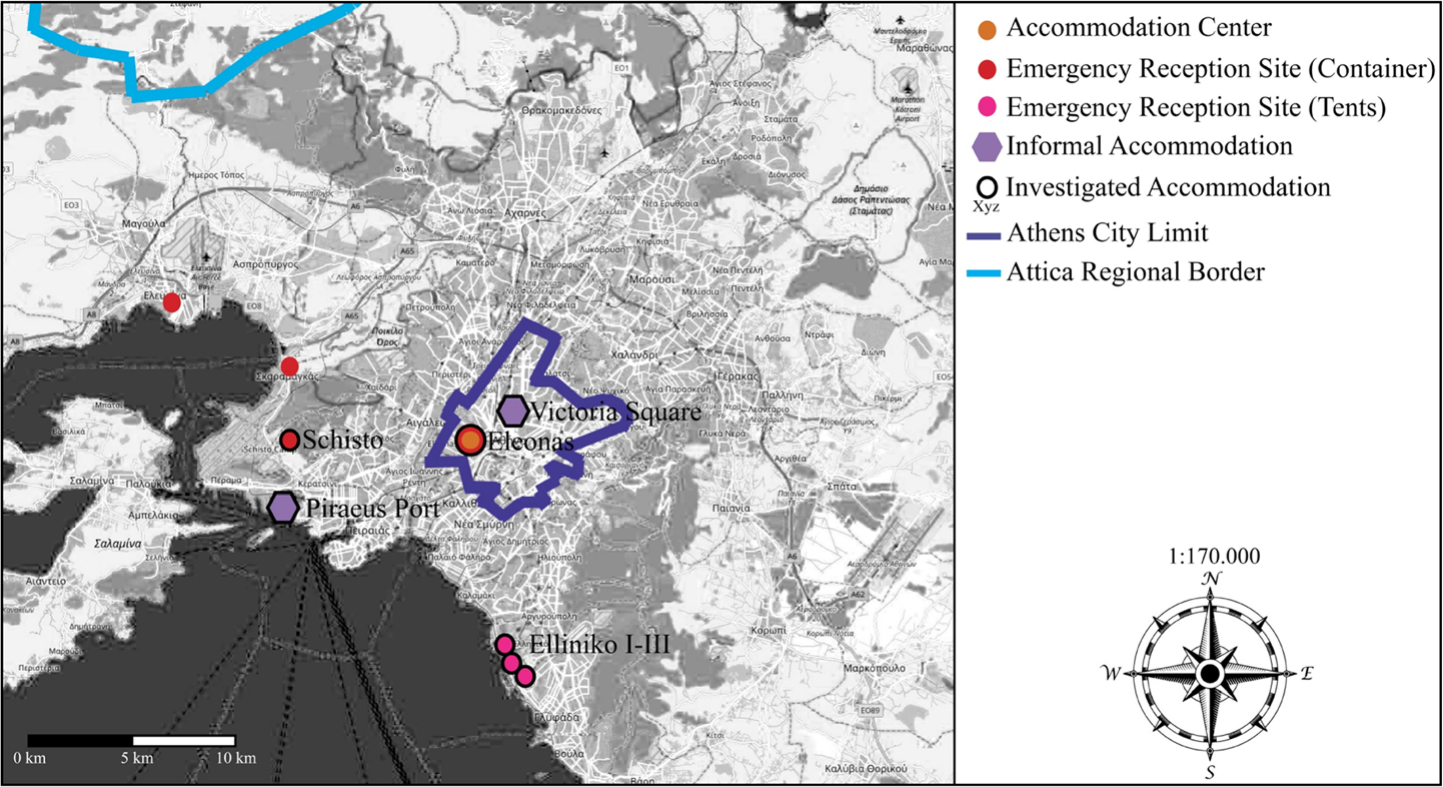
Schematic map of accommodation forms and their distribution in Greater Athens in 2016. The map was generated using OpenStreetMap.org. I modified the map by including data and information on accommodation, which are based on conducted interviews, own mappings and socio-spatial analyses, as well as on data provided by UNHCR (2016). Greece Factsheet 1–30 September 2016. https://data2.unhcr.org/en/documents/download/51901. Accessed 9 December 2017.

As a result, large-scale state accommodation has been developed (see Map 1): The Hospitality Centre Eleonas and the Emergency Reception Sites of Schisto and Elliniko I-III. These sites host people who have left the Greek islands and arrived on mainland Greece, and those who have returned from closed border areas. Eleonas is the only accommodation within Athens’ city limits, located in an industrial area. It houses some 2500 refugees. Schisto is an isolated settlement, located approximately 14 km from Athens, and has a capacity of 2000. Compared with Elliniko’s tent camps, Eleonas and Schisto both consist of containers including heating, water, and supply facilities. The Elliniko accommodation was built on Athens’ former airport (Elliniko II) as well as on the former Olympic hockey (Elliniko I) and baseball stadiums (Elliniko III), some 15 km from Athens. Around 4700 refugees live in Elliniko in 600 tents in or next to the halls of the buildings. It is the largest accommodation in the Attica region. There is no heating, and residents share 47 toilets and 120 showers.

The accommodation in Attica is secluded, fenced, and controlled. The Ministry of Migration Policy and the Hellenic Army run the operation, while the City of Athens additionally operates Eleonas. Nevertheless, state officials fail to provide basic humanitarian services in all five sites. Volunteers, activists, and local and international NGOs fill the gaps in humanitarian support. In interviews, the Hellenic Red Cross (2016), Solidarity Now (2016), and the Greek Council for Refugees (2016) have criticized Schisto and Elliniko in particular because of their isolated locations and the lack of basic living conditions.

### Berlin: Ambivalences of housing inclusion and exclusion

Contradicting German federal law on accommodation (AsylVerfG, §51(1)), in 2003, Berlin implemented an act (AV Wohn-AsylbLG) allowing housing in apartments after 3 months in a reception or accommodation center. Since 2011, there has been cooperation between six public housing companies and the senate in providing 275 apartments each year exclusively for asylum seekers and refugees. The LAF has developed a department that supports refugees in finding apartments. In 2009, around 80% of Berlin’s asylum seekers and refugees lived in private apartments. This proportion has changed dramatically since 2015 due to the increase of arrivals, tensions in Berlin’s housing market, and the city’s failure to provide affordable housing (Refugee Council Berlin, 2015). In 2016, around two-thirds of asylum seekers (37,000) were living in centers (Senatsverwaltung für Gesundheit und Soziales in RBB, 2016).

**Map 2:**
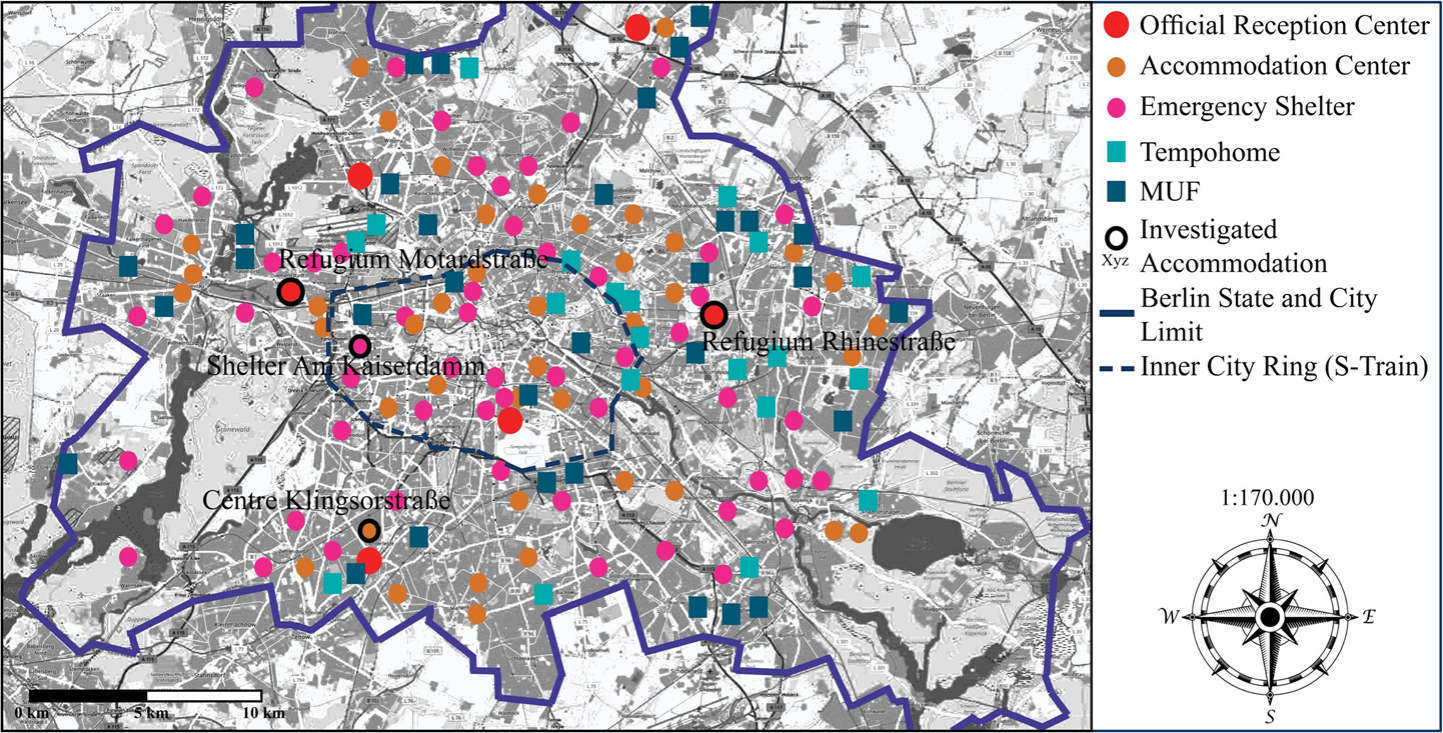
Schematic map of accommodation forms and their distribution in Berlin in 2016. The map was generated using OpenStreetMap.org. I modified the map by including data and information on accommodation, which are based on conducted interviews, own mappings and socio-spatial analyses, as well as on unpublished parliamentary documents of the Berlin Abgeordnetenhaus.

At the same time, the extent of refugee accommodation has grown from six centers in 2008 to more than 100 in 2016. Berlin’s decentralized accommodation system comprises a very diverse set of non-commercial and private operators and sites that are relatively equally distributed throughout the city (see Map 2). Three forms define the system according to their functions in 2016: Six official reception centers, 45 accommodation centers, and 66 emergency shelters. Emergency shelters have introduced a paradigm shift in Berlin’s refugee housing strategy: Since 2014, it has become the most common arrangement. Large halls and hangars–places not usually defined as housing–are divided into different segments by walls and tent structures and house up to 2500 people. They do not meet Berlin’s minimum reception standards.

The objective to develop mobile container or temporary homes (“Tempohomes”) and modular accommodation (“MUF”) since 2015, further manifests the city’s strategy of housing refugees in mass accommodation. Tempohomes are containers used for 3 years as an immediate reaction to the increase of arrivals. The 20 container villages consist of eight residential container complexes, one administrative building, one central supply building, and a gatehouse (LAF, 2016). The MUF has better conditions than the usual accommodation centers, but is also equipped and structured with fences, security guards, surveillance, and shared rooms. The five stories and prefabricated buildings house up to 500 refugees (Senatsverwaltung für Finanzen, 2016). Some 32 MUF have been built or are in the planning process. They will eventually house up to 24,000 refugees in total. Most MUF and Tempohomes are or will be located at the edge of the city and/or in industrial areas (see Map 2).

### Copenhagen: A “zero commune” barely accessible to refugees

**Map 3:**
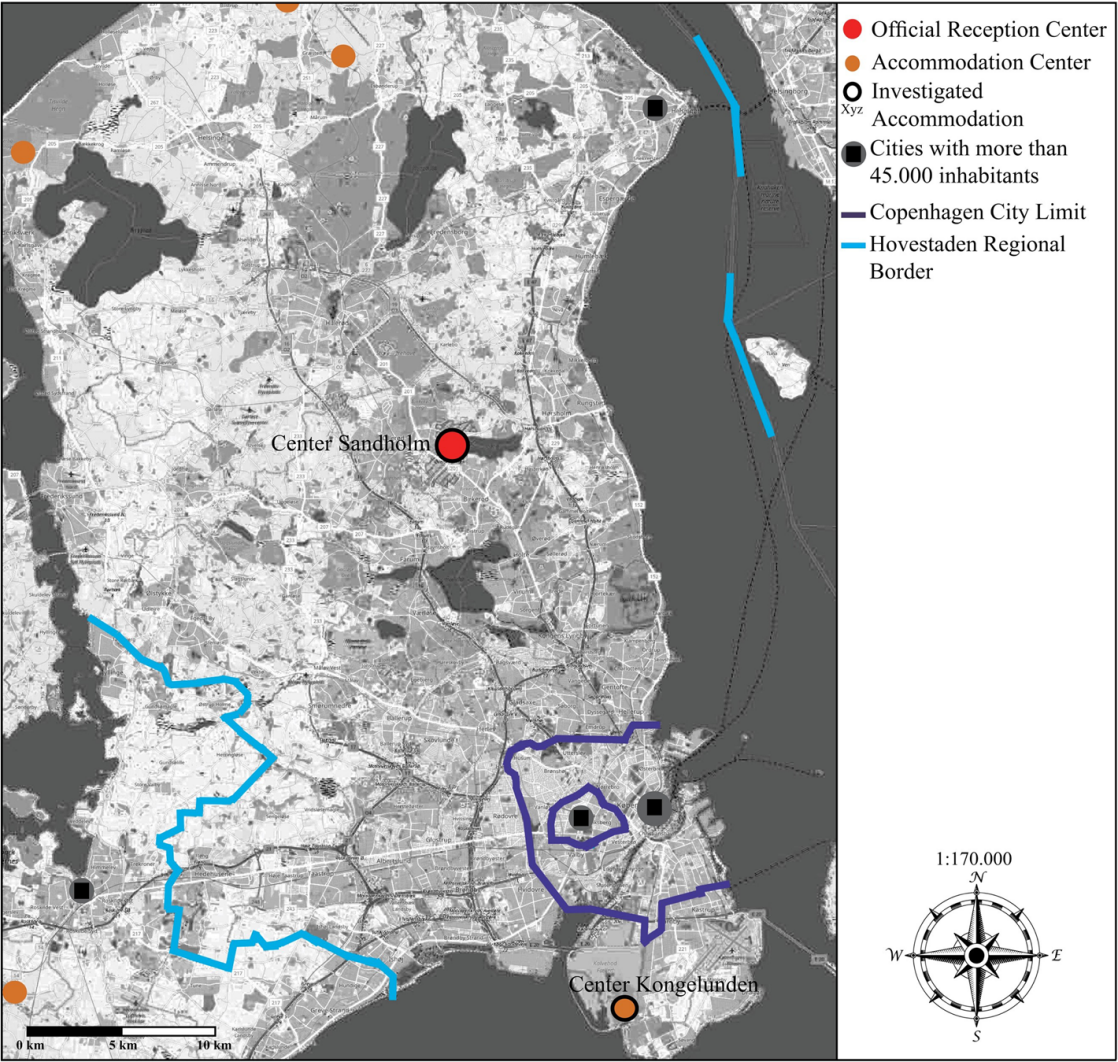
Schematic map of accommodation forms and their distribution in Hovestaden (without Bornholm) in 2016. I modified the map by including data and information on accommodation, which are based on conducted interviews, own mappings and socio-spatial analyses, as well as on data provided by Røde Kors (2016). Røde Kors Asylcentre. https://www.rodekors.dk/det-goer-vi/roede-kors-asyl/asylcentre. Accessed 9 December 2017.

Denmark’s Hovestaden region covers the greater Copenhagen area in the northeast of Zealand as well as the island of Bornholm.^3^ Copenhagen is its largest city. Hovestaden has a long tradition of accommodating asylum seekers. As illustrated in Map 3, most shelters are located remotely from any urban settlements. There are no centers in cities with more than 45,000 inhabitants. Centers are generally located in former military bases and hospitals in forests at least 10 but often up to 50 km away from larger urban settlements.

There are eight centers in Hovestaden (five on the mainland and three on Bornholm) and Center Avnstrup, which situated close to the region. The Sandholm center is a central landmark in the Danish system and in Hovestaden. The old, yellow military barracks were built in 1909 and opened in 1986 as an accommodation site. The center is located some 30 km north of Copenhagen. Sandholm reflects trends of centralizing several functions in one place, the standardization of services and spaces within the centers, accommodating large numbers, and of locating centers remotely. Its area includes sections for reception, accommodation, and departure. It houses the immigration section of the Danish National Police, the Immigration Service, and Ellebækhus; Denmark’s institution for detained asylum seekers operated by the Danish Prison and Probation Service. It accommodates up to 600 individuals, who are either newly registered, awaiting a decision on their application, or have had their application rejected and are awaiting departure from Denmark.

The distribution of centers is linked to Denmark’s objective to segregate asylum seekers in remote areas outside of urban settlements and to disperse accepted refugees to municipalities with a low immigrant population. The instruments for this policy are quota systems of the Immigration Service that regulate the allocation of accepted refugees to regions (*Regionskvoter*) and municipalities within the regions (*Kommunekvoter*). The number of residents in a municipality relative to Denmark’s total population, the number of immigrants in a municipality, and the number of reunified families in municipalities are used to calculate the quota (Danish Immigration Service, 2017). Large cities with relatively high proportions of migrant populations are termed “0-municipalities.” No refugee can move or be distributed to these cities, because “there are already too many immigrants in larger cities and that prevents integration” (Danish Immigration Service, 2014). This quota also affects the distribution of asylum centers to less populated areas with low proportions of immigrants. Since accepted refugees do not have access to larger cities, it would not be feasible to open centers in 0-municipalities (Danish Immigration Service, 2014). As a result, there is no asylum center in or in the neighboring municipalities of Copenhagen, because they are all 0-municipalities. Neither asylum seekers nor acknowledged refugees have the legal opportunity to live in Copenhagen and its suburbs.

## Objectives and characteristics of the Campization of refugee accommodation

In all the case studies, the 2015 “European Refugee Crisis” has stimulated the tightening of national asylum laws and local practices with regard to (but not limited to) accommodation addressing longer durations of obligatory stays in the camps (at least during the asylum application process), the enlargement and legal securing of mass accommodation, the opportunities to introduce remote locations for accommodation centers, detention practices during the application process, and legal exemptions for development, standards, and capacities. The current contribution illustrates the process of campization by parsing the causal relationship between the increase in the number of refugees, the introduction of a wave of new asylum laws, and reception conditions and the establishment of camp-like socio-spatial structures of accommodation. It argues that the objective of states and laws to produce stigmatized and excluded subjects is deeply realized in the space of campizated refugee accommodation.

### Stricter laws and their objectives

In Greece, asylum reforms are driven by EU regulations and the implementation of the EU-Turkey Statement. The 2013 and 2015 Greek Action Plan on Asylum and Migration Management, and the Law 4375/2016 (Nomos [Law] 4375/2016), concern the organization of the Asylum Service, the Reception and Identification Service, and the establishment of the General Secretariat for Reception, comprehensively reforming its “insufficient” asylum system (Commission Recommendation (EU) 2016/2256). The Law 4375/2016 provides a legal basis for the establishment of different accommodation facilities: Reception and identification camps, temporary reception facilities for asylum seekers (Article 8c), and temporary accommodation facilities for people “who are under a return, removal or readmission procedure” (Article 8d), with a maximum stay of 1 year. The law provided grounds for the development of temporary facilities with around 50,000 places in 32 camps in 2016 (Ministry of Interior, 2016), falling short of the requirements stipulated in the EU Reception Conditions Directive (Directive 2013/33/EU). Except for Leros and Eleonas in the Attica region–the only officially established state accommodation–the legal status and administrative responsibility for around 30 temporary facilities remains unclear.

In Germany, most changes have applied to the Act on the Acceleration of Asylum Procedures, but also to the Asylum Act and the Act on Changes in the Construction Planning and Zoning Law. The Act on the Acceleration of Asylum Procedures has (re-)introduced the benefit-in-kind principle. Refugees in reception sites only receive allowances in kind instead of cash benefits (AsylVerfG, Article 2 (3)). The period of the stay in a reception center has been extended from 3 to 6 months (AsylVerfG, Article 15a and AsylG §47) and asylum seekers with a low likelihood of being granted asylum must stay in a reception site until they receive a decision on their case. Article 30a of the Asylum Act (AsylG) introduced an accelerated procedure, which opens up the possibility to accommodate refugees with a low likelihood of being accepted in five special reception facilities nationwide. The Act on Changes in the Construction Planning and Zoning Law has affected the location and facilities of refugee shelters dramatically. Section §246, Articles 8–13 of the Federal Building Code (BauGB) now enables the location of accommodation in industrial and commercial areas, the exemption from building and use requirements, the conversion of office buildings and warehouses to refugee shelters, and the installation of mobile structures such as tents and containers. In Berlin, the new regulations have resulted in the development of Tempohomes, the conversion of warehouses, commercial and industrial buildings such as the former Tempelhof Airport to refugee accommodation, and the enlargement of emergency shelters, which, before the law changed, did not meet the minimum standards.

The amendments to the Danish Aliens legislation, L87, are aimed at conveying a message to make it “less attractive” to seek asylum in Denmark. Various measures that reduce benefits by 10% and force refugees to participate in covering costs (so-called “user charges”) characterize the act. Refugees must pay fees to apply for things such as family reunification, the extension of a residence permit, or an appeal against rejection. The new law (L87, Article 40 (9)) further allows police to seize asylum seekers’ assets to cover the costs of national assistance (accommodation, food, and health service). If an asylum seeker is in possession of adequate funds, the Danish Immigration Service will not provide state-financed accommodation. However, living in centers is mandatory until there is a decision on the asylum application. Refugees are thus forced to pay for a place in a state-financed center. The legislation further focuses on increasing the capacity for accommodation, including the introduction of state-operated tent camps as new forms of housing. To guarantee center housing, financial support for refugees who find their own housing has been cut. Another new item of legislation, L62, concerns immigration-related detention, introducing circumstances such as mass arrivals for detaining refugees.

These law changes introduced or enlarged the installation of centers with a large capacity and a longer detention time, with the purpose of securing the containment and territorialization of refugees. They include legal options that enable temporary or short-term facilities to become permanent sites without any improvement, and often with a worsening of conditions. More importantly, they are in line with and further perfect two major objectives inherent to the EU’s and nation states’ asylum politics: First, deterrence and reducing the number of arriving refugees, and second, the promotion of the “voluntary” emigration of refugees. The implementation of poorer living conditions, minimal welfare support, and obligatory center housing aim on the one hand to make living as hard as possible. On the other hand, these means are linked to the narrative of refugees being a burden to society and to the introduction of asylum austerity, which Darling (2016b) extensively discusses regarding the UK, as a result.

These objectives were the rationale behind introducing camps in the first place. In Germany, they were legally implemented along with the prominent statement by Lothar Späth (at that time Minister President of Baden-Wuerttemberg) in 1982: “the jungle drums in Africa shall spread the word: Don’t come to Germany, you will have to live in a camp here” (Späth, in Bozic, 1998, my translation). Berlin’s Refugee Council (2015) argued in an interview:

To this day, deterrence is the aim of the camps. In the social welfare law for asylum seekers, deterrence is the justification for restrictive asylum laws and minimal welfare support in general and for the development of camps in particular.

In Denmark, the objectives of deterrence and voluntary leaving are officially written down in the law L 87, section 1.2. The Danish Refugee Council (2014) stated:

They do not want to make them [the refugees] feel too comfortable, because they are afraid that more refugees would come and that they would stay permanently. It is a very nationalistic discussion on how to protect the Danish society.

In interviews in Greece, Athens’ city authorities (Papagianakis, 2016) and even NGOs justified center housing based on the current emergency state and the change from transition to destination, despite their general critique on the standards of the centers (Hellenic Red Cross, 2016; Greek Council for Refugees, 2016). However, the fear of refugees arriving “in masses” shows that Greece also resists providing solutions for the longer or even permanent stay of refugees.

### Intensifying encampment and its socio-spatial structures

The campization of accommodation in European city regions is reflected architectonically, functionally, and socio-spatially. Architectonically, the camp symbolizes a consolidated and secluded space separated from urban settlements. It can consist of several buildings, which when combined create the camp space or settlement. This is very visible in Centre Sandholm, Centre Kongelunden, Eleonas, Elliniko, and Schisto, as well as in Berlin’s Refugium Motardstraße, MUFs, and Tempohomes. The land is organized in a parcel-like configuration and marked by perimeters, usually in the form of fencing, walls, and a surveillance infrastructure. Built structures that are not suited for housing, such as containers (Berlin, Copenhagen, and Athens), tents (Athens), and commercial and industrial buildings (Berlin, Copenhagen, and Athens) further characterize the camps. These characteristics create a clear distinction between the inside and outside, and label the camp as an abnormal form of housing a group. Another tendency that the cases illustrate is the notion of centrality within the camp space. In Centre Sandholm and Centre Kongelunden, Berlin’s Motardstraße, and Eleonas, the basic infrastructures (canteens, laundry facilities, clothing distribution, administration and consultation, leisure spaces) are usually located centrally at the entrance of the camp or in the middle, surrounded by the residential buildings.

Functionally, campization results in blurring the differentiation of the classic functions of European refugee accommodation: Reception, accommodation, and detention. Centre Sandholm and Centre Kongelunden, and some facilities in Berlin and Athens show a concentration of these functions in one place. Thus, an official registration and reception site may also be a place where long-term accommodation and deportation is conducted. An accommodation center that is set up to house asylum seekers during the application procedure may easily become a place of deportation. The concentration of multiple functions, however, is reflected in the functional segregation of the camps. In Centre Sandholm and Ellinko, for example, there are various sectors and zones that constitute spaces of first reception, long-term accommodation, and deportation within one camp. Furthermore, campizated accommodation tends to consist of its own infrastructures and the facilities for daily needs (such as schools, clinics, playgrounds, canteens, and wash houses). The cases of Berlin’s Motardstraße, Hovestaden’s Sandholm and Kongelunden, as well as the camps in Attica illustrate that different usages of space and daily routines are functionally segregated into various zones.

With regard to socio-spatial characteristics, encampments illustrate the social differentiation and segregation processes for the residents. In Centre Sandholm, Eleonas, and Ellinko, officials and center staff group residents in various blocks and units according to their legal status (i.e. asylum seekers, acknowledged refugees, people entitled to subsidiary protection, and rejected asylum seekers) as well as characteristics concerning race, ethnicity, gender, age, and family status. This creates a hierarchy of power, which applies, first, to the relation between center staff, who allocates refugees according to these characteristics, and residents, who must accept this form of distribution. Second, it results in a hierarchy and envy between residents, because of the strong socio-spatial divisions regarding their legal positions and because residents with a low likelihood of being granted asylum are usually housed in less equipped facilities. The spatial configuration of the camp space and socio-spatial concentrations reflect these hierarchies. The expansion of control mechanisms further characterizes campization. All the camps in Greater Copenhagen and Athens have identity and access control as well as security guards (often in the form of the military). The control by the staff over the actions conducted in the camp, including the intrusion of privacy when entering a room of a refugee, further shapes social interactions. Control is an inherent part of camps and refugees generally “live under forms of control that do not apply to other citizens” (Pasquetti, 2015, p. 704).

There are differences in the quality of campization in the case studies. The differences are in relation to state laws and local practices concerning accommodation and generally the law and social benefit contexts they are a part of. In Attica, campization is very pronounced because of Greece’s transformation to a destination, its challenges in accommodating refugees permanently, and the lack of an elaborated, legally binding, and differentiated asylum and accommodation system with clear standards and functions. Nevertheless, the campization of accommodation is apparent in all the case studies. The investigated cases generally illustrate the tendency to locate refugee accommodation in secluded areas outside of cities, and of governing these spaces by legal instruments that do not apply to surrounding areas and people. The camp signifies a state of exception that is increasingly normalized and that has intruded European accommodation. Even though the camps are theoretically open, the mentioned characteristics establish a closed character, which aggravates the mobility between the inside and the outside of a camp as well as-due to inner segregation processes–even within a camp.

### Campization and the states’ objective to produce stigmatized subjects

Accommodation practices are material realizations of asylum laws, and refugee accommodation illustrates the physical space of administrative and political acts of power. This space is politically developed for the purpose of separating the “own” and the “(ethnic) stranger”; citizens and non-citizens. It is also a space between. A space where refugees belong neither here nor there and thus it is a space where “refugees challenge the assumed link between nations, state and citizen” (Turner, 2016). The refugee camp is developed in a state of emergency, a spontaneous solution to accommodate high numbers of migrants. However, more importantly it attempts “to contain ‘matter out of place’ that refugees constitute and [to] re-stabilize the national order or things” (Turner, 2016).

Because of this attempt, the refugee camp fulfils three objectives that are necessary to secure national order. First, the space of the accommodation guarantees that refugees remain subjects of the state. On the one hand, they are excluded and put into exceptional places, which have been legally developed to house this particular group and not citizens. On the other hand, they are included in the structures of government and national laws. Second, the development of camps is a result of the use of force and exclusive alien acts in the first place. Camps are embedded in laws, which have been developed out of racist discourses in Europe (Wichert, 1994). They are thus a central element of what Pieper (2008)-regarding accommodation in Germany–argues is “institutional racism.” The development of the camp is a product of racist laws (i.e., the production of space through racism). Third, because of the allocation of refugees to a negatively connoted space, they become visible, which is the starting point for processes of racialization and “territorial stigmatization” (Wacquant, 2007). Housing in a camp produces attention due to the material and inner structures of the camp space and it simultaneously degrades those who live in it. To the outside world, the camp conveys an image of residents who do not accord with societal norms and who deserve to live like that. These conditions result in a reinforcement of unfounded fears and racist attitudes (i.e., racism through the production of space).

State-organized camps, however, are not only top-down institutions, but are also shaped and influenced by the socio-spatial practices of the people who live in and use the accommodation. Refugees experience life in accommodation in different ways. The effects of asylum laws and of the refugee status on people “can differ radically from context to context, from person to person” (Malkki, 2002, p. 358). Refugees can experience protection or exclusion, support or discrimination, or something else; possibly all at the same time. Within my research, I have tried to respect the challenges of studying and the danger of generalizing refugees’ complex experiences. I identified three factors that impact the majority of refugees interviewed in the case studies.

First, refugees highlight the problems of the location and the physical structures of camps. They state that they are labeled as criminals, as others, and abnormal to the outside. Because of their architecture and structural organization, location, and symbolism, the camps play a crucial role in the formation of resentment toward “strangers.” A refugee in Hovestaden stated:

The location of the asylum center helps to establish an image of refugees as being criminal and that we are scary. This image is taken over by some parts of society. Danes thus get the picture that we are too many and that we are causing problems (Refugees in Denmark, 2016).

Sandholm, and also the centers in Greater Athens are the most obvious examples of the camp architecture representing a place of stigmatization, intimidation, and otherness. One refugee, who lived in Sandholm, remembered:

For me, the problem in Sandholm is that the military site is next to it. Every morning they start firing practice right next to people who have escaped from war. This is disgusting (Refugees in Sandholm Denmark, 2014).

Second, interviewed refugees highlight the role of control and dependency in the accommodation and in creating daily routines. The (re-)introduced principle of allowance in all the case studies and the decrease of welfare support reinforce dependencies toward staff and social workers. Heteronomy–the lack of autonomy for refugees to arrange their life–already starts on arrival and is a fundamental part of the asylum procedure. It is linked to the distribution to a center, to a room, to a bed, to fixed meal times, and to scheduled access to common rooms. A person who lives in Berlin’s Motardstraße highlighted some of the dependencies he deals with:

If you want to wash your clothes, you need an appointment. If you want to change your bed, you must ask them … They choose the appointment. And one time, I was in the cab. I wanted to meet someone and bring him to my home. But it was not possible to bring him to the camp because after 10 p.m., you cannot bring strangers. The security does not allow it … You cannot really do anything without asking somebody else … I cannot cook for myself, but I want to. However, there is no kitchen and we also only get little money (Refugees in Berlin, 2015).

These characteristics also affect social interactions, which are characterized by control, hierarchies, and the lack of privacy. Due to the forced placing, conflicts may emerge that influence individual daily routines. Administrative bodies often concentrate refugees from the same ethnic background, which causes problems if the refugees do not share a religion, ideologies, interests, etc. A refugee, who lived in Refugium Rhinestraße in Berlin, explained the difficulties that can arise from this approach:

At first, I was alone in my room. Then one Pakistani came and then another one. But they were drinking and smoking all the time. I was afraid of them and I always had to hide who I am … I was wearing shorts and a guy said: “Are you Muslim?” I replied “Yes.” And he said: “Why are you wearing this? Who are you? Are you gay?” (Refugees in Berlin, 2016).

Third, it is the period for which people need to live in the camp that affects their experiences. Many of the interviewed refugees stated that after their arrival, they felt mostly pleased to experience protection and to have a place in a center. However, after some time, refugees feel that the accommodation limits their lives. Kublitz (2016, p. 229) concludes in her studies on Palestinians in Danish camps that “life in the Danish camps is characterized by minor mundane catastrophes that are each so small that they barely register or elicit a moral response, but nevertheless erode the lives of my interlocutors.” An asylum seeker and activist confirmed this finding referring to Hovestaden:

The camps here are like in paradise in the beginning. The conditions are better than in Africa. But the personal situation they put the people in is very bad … After a while, you want to do things. You want to start a normal life, because you don’t want to live forever like this … The asylum center is like a concentration camp. The only difference is that they don’t burn people. They let them gradually die (Refugees in Sandholm Denmark, 2014).

The law changes of 2015 and 2016 introduced longer obligatory durations in the refugee accommodation. Since this is linked to cuts in welfare, bans from the labor market, and from education, it often develops into a waiting zone, a permanent temporality (Hailey, 2009), reinforcing conflicts, processes of exclusion, and social and mental problems. A refugee in Eleonas camp (2016) depicted this state:

I can do nothing. It is boring. Waiting. I sit outside sometimes. Waiting … A lot of people have problems sleeping. I only sleep 2 to 3 hours per night … We came here normal, with problems, yes, but normal, and now we are crazy. We have problems, but they do not help us. Instead, we are pushed outside of the cities into the countryside (Refugees in Athens, 2016).

The case studies also show that camps are not solely excluded and isolating areas. People live in them, work in them, carry out voluntary and social work, and visit residents across their boundaries. Despite the stated closed character, in all the case studies refugees leave the site in search of engagement with the camp’s socio-spatial environment. The rise of voluntarism and the support of civil society actors further illustrate the links between a site and its surroundings. Refugees find other ways to become a part of the society and liberate themselves from camp life. Strategies may include living officially in state accommodation but staying with friends in the city, working in the informal economy, protests, “going underground,” or developing a center council to represent the interests of residents. An activist with a refugee background in Copenhagen cites an example, which outlines trends I observed in all cases:

Some of them [refugees] have a black job. I know all of them. In 1991, I came here as a refugee. But if people don’t get a black job, they are not able to pay for transport and for access to the city. That is why they do little businesses (Refugees in Denmark, 2016).

These informal practices (Sanyal, 2012) often include refugees actively shaping space through spatial appropriations. In the Attica region and Berlin, poor living conditions resulted in the development and enlargement of informal sites such as Victoria Square and Piraeus Port, as well as Oranienplatz and Gerhard-Hauptmann-Schule between 2014 and 2016. Refugees interviewed in Athens explained that they preferred to stay at these informal sites because they experienced a form of autonomy. Around Victoria Square and particularly in the Athens neighborhood of Exarchia, activists, local initiatives, and refugees have occupied empty buildings such as hotels in order to house refugees. In 2017, more than 2500 refugees and migrants were living in these squats (Chrysopoulos, 2017). The main structures of civil support and NGOs are located around the square, having transformed the area into a *Neighborhood of Arrival*.

The Oranienplatz in Berlin and Victoria Square in Athens have also been major sites for refugee protests and political actions aimed at raising attention for their deprived situation and the poor housing conditions. In the Copenhagen region, there is a “Close the Camp” demonstration that takes place on occasion. The organization “Grandparents for Asylum” has been demonstrating against the bad living conditions in the camps every second Sunday of each month in front of Sandholm.

Despite the emergence of measures that “close” the accommodation, the theoretical openness is the major difference from prisons, prisoner-of-war camps, or closed refugee camps. It is to some extent a “porous institution” (Kreichauf, 2016, p. 200). Political activism, strategies of survival, and reclaiming autonomy may emerge out of and/or because of the camps. Studies on camps in the global south show that they are not only places of “bare life” (Agamben, 1998), but that they are also places where new social forms and formations (Corbet, 2016; Lecadet, 2016), a distinct political life (Ramadan, 2013), a site of politics, “urban practices” (Sanyal, 2014, p. 568), resistance, and identify formation (Malkki, 1992) emerge.

## Conclusion

In this contribution, I argue that another wave of rigorous laws and policies have impacted on the development of large camp-like mass accommodation with poor housing standards and aggravated forms of confinement. I have introduced the term campization to, first, elucidate the socio-spatial changes and characteristics of refugee accommodation in European city regions, second, to offer ways to understand through what processes and rationales these features emerge, and third, to strategically emphasize the trend of adapting camp characteristics, which are usually associated with camps in the global south.

The legal stabilization of permanent, enlarged, obligatory, and spatially isolated camps with increased capacities, and their functions to territorialize, marginalize, contain, and deter immigrants constitute campization. In the empirical reality, there is a “diversity of refugees and camps” (Sanyal, 2012, p. 634), and the campization of refugee accommodation may take various forms depending on the camp’s integration in legal frameworks, the socio-political functions and, of course, the specific characteristics of single sites. However, particular architectural, functional, and socio-spatial determinations outline the investigated camps.

The camp’s nature is to separate populations, contain a specific category of people and to territorialize immigrants in extraterritorial locations. Camps are demarcated and, especially due to recent law changes, exceptional in legal terms. They mark the refugees’ position in society. They are excluded spatially and legally on the one hand, and on the other, are “defined and contained by the surrounding society” (Turner, 2016, p. 142). The camps have been institutionally established, because they follow the political objectives to protect the EU’s and national orders and to deter migrants. This is in line with the EU’s general attempts to reduce the number of refugees and to extraterritorialize migrants inside and outside of the EU.

On the one hand, the process of campization is a material expression of the stabilization of a state of emergency and temporality. The camp becomes permanent; it is a space of “permanent temporality” (Hailey, 2009). Changes in acts, regulations, and building codes are manifest in the camps’ spatial structure and objectives. On the other hand, this material stabilization of temporality reinforces the objective of seeing refugee migration as a phenomenon limited in time. A camp becomes a permanent place to regulate immigrants, but the immigrants can only stay temporarily in a spatially confined way. This is reflected in the states’ attempts to make access to family reunification, permanent residency, or even citizenship more difficult. A lot of resources are instead directed to campaigns and state programs promoting “voluntary return.”

What does campization tell us about the transformation of migration and urban development processes, arrival infrastructures, and asylum? First, campization points out trends to territorialize immigrants for longer periods, even if they have been granted protection. The Danish Kommunekvoter determines the dispersal to municipalities of refugees granted asylum. Germany’s residential obligation (§ 12a AufenthG, Bundesministerium der Justiz und Verbraucherschutz, 2016) allocates accepted refugees to municipalities for 3 years. In both cases, political authorities aim to prevent the development of migrant concentrations in larger cities. Studies on other migrant groups show that such acts are the “breeding ground for the forced and politically induced concentration and disintegration” of immigrants on the local level because of deflective and discriminative local policies, which aim to edge migrants away from central parts of the allocated city (Kreichauf, 2015, p. 20). The camp is a starting point for the state to territorialize refugees. The forced confinement on arrival is reproduced in the distribution of refugees once they are given asylum, limiting their physical and social mobility for longer periods.

Second, the characteristics of camps in Europe also highlight that (refugee) migration is deeply related to discourses on crime, terror and a general criminalization of migration (Wacquant, 2012). “Refugees, asylum seekers and undocumented migrants are often represented as … a security threat to nation-states that must be restricted by a violent and repressive geography of walls, coastguard patrols, detention camps and offshore processing” (Ramadan, 2013, p. 65). Borders and camps are sites through which these logics materialize (Maestri & Hughes, 2017). Controls and closures at and of borders have restricted free movement within the EU over recent years, while the EU and its member states have further sealed external borders. Borders and camps are devices of this “crisis of free movement” (Hansen, 2017; Lillie & Simola, 2016), and are often interweaved in terms of their spatial and functional rationales. In the age of terror, there is moreover a growth of urban anti-terrorism measures such as structures for security and surveillance, and physical barriers. The camp can be conceptualized as one of these measures, as a symbol of the campization of urban territory or of a camp-border urban development (Diken & Laustsen, 2005).

Third, campization reveals trends of neoliberalizing asylum (Darling, 2016a) and asylum austerity (Darling, 2016b). Mantras of cutting social benefits and the refugees’ contribution to financing the reception system increasingly characterize asylum. Camp operators, security firms, container companies, etc. have developed to important internationally acting, non-state, and profitmaking actors in the asylum arena. The transfer of tasks from public authorities to profit-seeking companies directly unfolds in the camp’s structures and the erosion of living standards. Asylum austerity and campization introduce and secure substandard living as a durable norm, not only for refugees. In Berlin and in Hovestaden, some facilities have been built to eventually house homeless people, students, the elderly, and lower-income populations. Refugee accommodation as such can be a perfect arena for neoliberal experimentation reacting to marginalized populations and also in “wider areas of social housing and social care” (Darling, 2016b, p. 500). The structures of refugee accommodation as places of confinement, substandard housing and a forced concentration of deprived groups thus may penetrate the structures of the urban.

## Footnotes

1 In this contribution, I mostly study the situation of refugees who arrive in an EU member state and apply for asylum “on the ground”; so-called asylum seekers. In legal terms, there is a distinction between a refugee, an asylum seeker, an internally displaced person, and a stateless person; as well as between those with different protection statuses (such as a person under subsidiary protection, etc.). However, I apply a general definition of the term “refugee” if I am not explicitly referring to a legal status group: A refugee is a displaced person who has been forced to leave her or his home country and who cannot return to her or his home safely. This definition includes the groups mentioned.

2 I use the terms Athens, Berlin, and Copenhagen to include their regions.

3 The centers and living conditions on Bornholm have not been further investigated within the research material presented.
